# What intrinsic factors influence responsiveness to acupuncture in pain?: a review of pre-clinical studies that used responder analysis

**DOI:** 10.1186/s12906-017-1792-2

**Published:** 2017-05-25

**Authors:** Yu-Kang Kim, Ji-Yeun Park, Seung-Nam Kim, Mijung Yeom, Seungmin Lee, Ju-Young Oh, Hyangsook Lee, Younbyoung Chae, Dae-Hyun Hahm, Hi-Joon Park

**Affiliations:** 1 Department of Science in Korean Medicine, Graduate School, Kyung Hee University, 26 Kyungheedae-ro, Dongdaemoon-gu, Seoul, 02447 Republic of Korea; 2 College of Korean Medicine, Daejeon University, 62 Daehak-ro, Dong-gu, Daejeon, 34520 Republic of Korea; 3 College of Korean Medicine, Dongguk University. 32, Dongguk-ro, Ilsandong-gu, Goyang-si, Gyeonggi-do 10326 Republic of Korea; 4 Acupuncture & Meridian Science Research Center, Kyung Hee University, 26 Kyungheedae-ro, Dongdaemoon-gu, Seoul, 02447 Republic of Korea; 5 Department of Acupuncture and Moxibustion, College of Korean Medicine, Kyung Hee University, Seoul, 130-701 Republic of Korea

**Keywords:** Acupuncture, Responder, Non-responder, Individual difference, Cholecystokinin

## Abstract

**Background:**

Not many studies have investigated individual sensitivity to acupuncture. To explore the intrinsic factors related to individual responses to acupuncture, we reviewed published pre-clinical studies using responder analysis on pain.

**Methods:**

We searched the PubMed and EMBASE databases to June 2015. We included pre-clinical reports describing responders and non-responders to anti-nociceptive and analgesic effects of acupuncture in animal study. We identified the potential intrinsic factors which might be related with the response to acupuncture.

**Results:**

Totally, 216 potentially relevant articles were retrieved and 14 studies met our inclusion criteria. Rat (*n* = 1348) and rabbit (*n* = 56) were used, and only electroacupuncture (EA) was applied as an intervention. Results showed that high levels of cholecystokinin-8 and receptors were associated with poor responsiveness to EA. Endogenous opioids including *β*-endorphin and met-enkephalin, descending inhibitory norepinephrine and serotonin system, and hypothalamic 5′-AMP-activated protein kinase seemed to be associated with high-level responses. Spinal levels of neurotransmitters and pro-inflammatory cytokines were also differentially expressed depending on the EA sensitiveness. In the central nervous system, hypothalamus, periaqueductal grey, pituitary gland, and spinal cord were suggested to be involved in the EA responsiveness. Identified individual variations did not seem to be accidental, as the responsiveness to EA was replicated over time. However, methodological issues such as reproducibility, cut-off criteria, and clinical relevance need to be further elaborated.

**Conclusion:**

Our study suggests that the identification of the biological factors differentiating responders from non-responders is necessary and it may aid in understanding how acupuncture modulates pain.

**Electronic supplementary material:**

The online version of this article (doi:10.1186/s12906-017-1792-2) contains supplementary material, which is available to authorized users.

## Background

Pain is the most prevalent reason patients seek medical attention in many developed countries [1, 2]. It is a chief complaint in numerous disorders that hinders the individual’s daily activities and functions [3, 4]. Analgesics can help alleviate such pain but individual sensitivity to pain and individual responsiveness to pharmaceuticals vary considerably even among patients with the same conditions. In the clinic, for example, although morphine has great analgesic properties, some patients experience inadequate analgesia and demand increased dosage or concurrently suffer from significant side effects [5]. These inter-individual differences in the factors causing pain and in the pain mechanisms per se, are clearly in play and a more individualistic approach, which focuses on the responder and non-responder differences, may be useful in the development of novel analgesic treatment [6–8].

Acupuncture, the stimulation of specific acupoints on the body with acupuncture needles, is widely used to relieve persistent pain and to treat many disorders [9–12]. An important feature of acupuncture is that the chosen acupoints and the preferred type of manipulation reflect patients’ individual characteristics and/or those of their diseases. Intrinsic patient factors are, therefore, at the forefront when doctors plan overall treatment [13].

For decades, researchers have tried to identify what intrinsic factors may constitute the specific effects of acupuncture. A recent innovative brain imaging study showed that acupuncture-induced analgesia of migraine involves the intrinsic functional connectivity of the right frontoparietal network [14]. However other than this study, most of the previous research has only focused on elucidating acupuncture’s therapeutic effects, while individual responsiveness was less considered [15]. Analysis of both responders and non-responders would reap the benefits additional to investigating acupuncture outcomes and developing therapeutic effects.

A few preclinical or human studies have provided significant insight into the differences between responders or non-responders. Electroacupuncture (EA) afforded either excellent or almost no effects in human or animals, i.e., it produced beneficial effects in approximately 70% of subjects and resulted in little changes in the other 30%. [16–18]. Low responsiveness to EA effects was known to be associated with changes in cholecystokinin (CCK) levels [19]. It was also suggested that the degree of responsiveness to acupuncture effects was associated with different neural activation by EA in periaqueductal grey (PAG) or hypothalamus [20, 21]. Thus, further work on the mechanisms involved and specific regulation of intrinsic factors related with high or low responsiveness might allow the therapeutic effects of acupuncture to be better predicted. However, to date, no systematic study has distinguished between responders and non-responders when acupuncture was used to modulate pain.

In this review, we reviewed the relevant published literature of pre-clinical studies to define high and low responsiveness of EA to pain and identify trends in the associated factors, regions, or mechanisms of responders and non-responders.

## Methods

### Search strategy

We searched the PubMed and EMBASE databases to June 2015. The search term was “acupuncture and (responder* or non-responder*).” Additionally, publications in the reference lists of retrieved papers and relevant reviews were manually retrieved.

### Study selection

We first screened the records by title and abstract; we included original English-language reports on animal experiments discriminating responders from non-responders. The exclusion criteria were: 1) use of a language other than English, 2) irrelevance in terms of acupuncture treatment, 3) lack of baseline or outcome data, 4) definition of responders using only a survey or a questionnaire, 5) the absence of any distinction between responders and non-responders to acupuncture, 6) the absence of a focus on differences between responders and non-responders, and 7) not conducted in animal models. We included any form of acupuncture and any animal model. As we sought to define the features of acupuncture associated with a response or a non-response, all included studies had to distinguish between responders and non-responders. Any ambiguities were discussed by three reviewers (Kim YK, Park JY, and Park HJ) until unanimous consensus was attained.

### Data extraction

We recorded the first author’s name, the year of publication, the country in which the work was performed, the animal used (sex and species), the sample size, whether the animals were healthy or diseased, the type of acupuncture applied, the EA parameters (if relevant), the acupoints used, the cut-offs for identification as responders/non-responders, the numbers of responders and non-responders, the outcomes of any behavioral tests applied, target molecules, regions of interest, the defined functions of target molecules, and the outcomes of EA-sensitivity analysis. Data extraction was performed by one author (Kim YK), but two other authors (Park JY, Park HJ) double-checked this work.

### Quality assessment

The included publications were independently assessed by two authors (Kim YK, Park HJ) in terms of the methodological quality using the 9-item checklist modified from the CAMARADES checklist [22, 23]: (1) peer-reviewed publication; (2) detailed statement of acupuncture procedure; (3) objective behavior test applied to classify responder or non-responder; (4) statement of cut-off values for dividing responder or non-responder; (5) notification of the ratio of responder or non-responder to the total; (6) blinded assessment of outcome; (7) reproducibility of EA sensitiveness; (8) compliance with animal welfare regulations; and (9) statement of potential conflict of interests. Each study was recorded a sum quality score out of a possible total of 9 points. Discrepancies were resolved after discussion between the two authors (Kim YK, Park HJ).

## Results

### An overview of the study

**Fig. 1:**
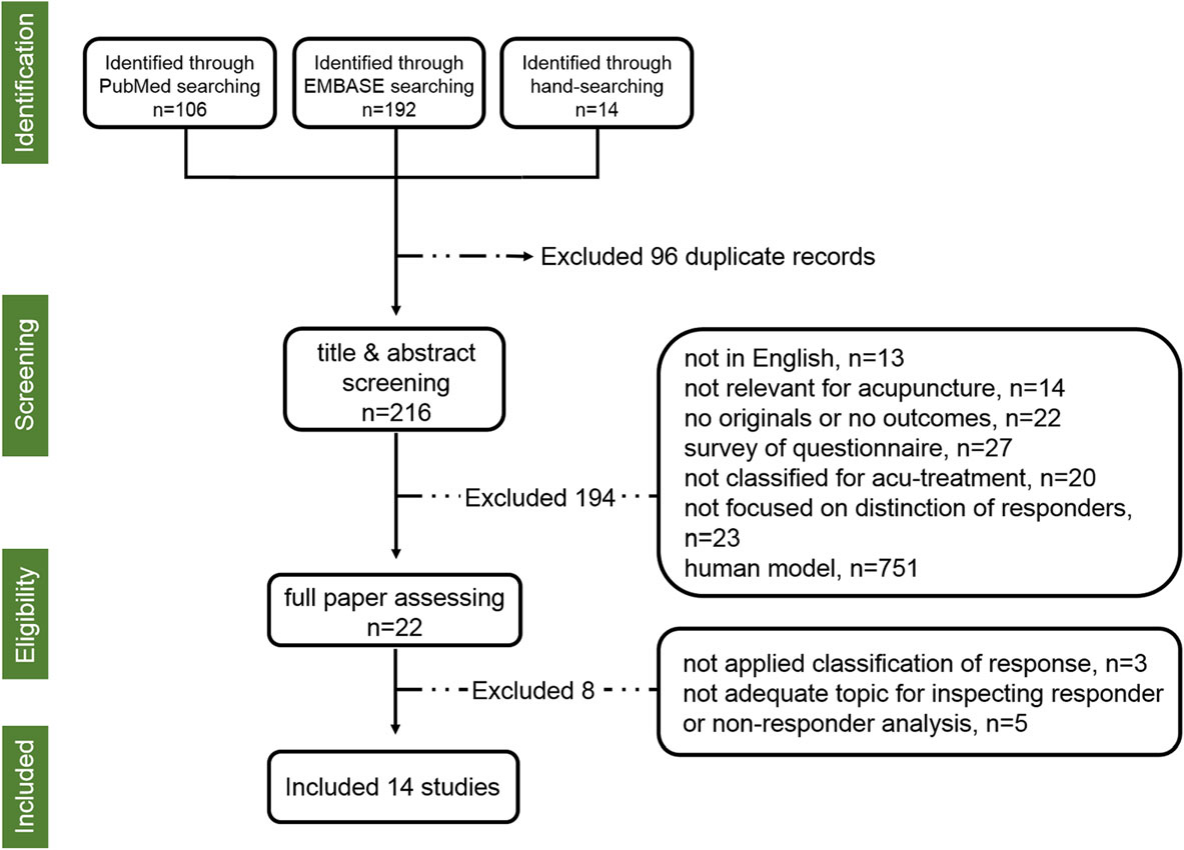
Flow diagram of the review

Figure 1 shows how reports were selected. We initially identified 106 and 192 relevant publications in PubMed and EMBASE respectively, and additionally added 14 works via manual searching. Among these 312 records, 96 duplicated articles were removed. We excluded 194 of these 216 papers after screening the abstracts and titles. Ultimately, 22 publications were fully evaluated. We subsequently excluded eight of these because responders were not classified (three studies) or studies did not explore factors associated with individual variations (five studies). Finally, 14 studies met our inclusion criteria.

### Characteristics of included studies

Table 1 lists the characteristics of the included studies. A total of 1348 rats and 52 rabbits were used. All studies featured EA of low frequency (1–3 Hz, nine studies), medium frequency (15 Hz, one study), or high frequency (100 Hz, five studies). All studies electrically connected acupoint ST36 with acupoint SP6 (six studies), or the adjacent bilateral or ipsilateral ST36 acupoints (six studies). One study used acupoint TE18 in the rabbit. Only three studies included pain models including neuropathic pain [18], incisional pain [24], and inflammatory pain [25] in their evaluations of pain levels; 13 studies employed normal animals.

**Table 1:** Experimental index for electroacupuncture

Author (Year, Country)	Animal (sex)	Sample size	Condition	Acupoints (side)	EA Parameters: frequency, amplitude, duration
Kim (2014, Korea)	rats (M)	18	Nor	ST36-subST36 (n.r.)	2 Hz, 0.2–0.3 mA, 20 min
Wang (2012, China)	rats (M)	170	Nor	ST36-SP6 (B)	2 Hz or 100 Hz respectively, 0.5/1.0/1.5 mA, 30 min
Fais (2012, Brazil)	rats (M)	48	Incisional pain	ST36-SP6 (B)	2 Hz, 1.4–1.5 mA, 20 min
Gao (2007, China)	rats (M)	18	Nor	ST36-subST36 (B)	1 Hz, 3.5–5 V, 60 min
Kim (2007, Korea)	rats (M) CCK-AR KO rats (M)	38	Nor / Neur. pain	ST36-subST36 (n.r.)	2 Hz, 0.2–0.3 mA, 20 min
Ko (2006, Korea)	rats (M)	12	Nor	ST36-subST36 (n.r.)	100 Hz, 0.2–0.3 mA, 20 min
Sekido (2003, Japan)	rats (M)	95	Nor / Paw inflam	ST36-subST36 (L)	3 Hz, 1/2/3 mA, 60 min
Lee (2002, Korea)	rats (M)	12	Nor	ST36-subST36 (n.r.)	2 Hz, 0.2–0.3 mA, 15 min
Liu (1999, China)	rats (F)	19	Nor	ST36-SP6 (B)	100 Hz, 1/2/3 mA, 30 min
Tian (1998, China)	rats (F)	193	Nor	ST36-SP6 (n.r.)	100 Hz, 1/2/3 mA, 30 min
Tang (1997, China)	rats (F)	215	Nor	ST36-SP6 (B)	100 Hz, 1/2/3 mA, 30 min
Takeshige (1993, Japan)	rats (M) and rabbits (n.r.)	402 rats and 52 rabbits	Nor	Rats: ST36 (n.r.) Rabbits: TE18 (n.r.)	1 Hz, intensity to cause muscle contraction, 30 min, 45 min or 60 min
Takeshige (1992, Japan)	rats (M)	80	Nor	ST36 (n.r.)	1 Hz, intensity to cause muscle contraction, n.r.
Han (1985, China)	rats (n.r.)	28	Nor	ST36-SP6 (n.r.)	15 Hz, 3 V, 10 min

*B* bilateral, *CCK-AR KO* cholecystokinin A receptor knockout, *F* female; Incisional pain, mechanical hyperalgesia induced by 1 cm longitudinal incision through skin and fascia and stitches on right hind paw; *M* male, *L* left; *Neur. pain* neuropathic pain; *Nor* normal, *n.r.* not reported; Paw inflam, carrageenan-induced inflammation on the paw; subST36, 5 mm distal from ST36

### Study quality

The quality score of the included studies ranged from 2 to 7 out of a total 9 points. Of the 14 studies, 3 studies got 2 points [19–21], 3 studies got 3 points [17, 26, 27], 3 studies got 4 points [28–30], 1 study got 5 points [31], 2 studies got 6 points [24, 25], one study got 7 points [18], and one study got 8 points [32] (Table 2). All 14 studies were published in peer reviewed journals. Six studies described detailed acupuncture procedure [24–26, 28, 30, 32]. Six studies reported objective behavior test applied to classify responder of non-responder [18, 25, 27, 29, 31, 32]. All 14 studies notified cut-off value for dividing responder or non-responder. None of the studies mentioned blinded assessment of outcome. Two studies confirmed reproducibility of EA sensitiveness [18, 32]. Six studies reported compliance with animal welfare regulations [18, 24, 25, 28, 31, 32]. Four studies contained statement of potential conflict of interests [18, 24, 31, 32].

**Table 2:** Risk of bias of the included studies

Author (Year)	A	B	C	D	E	F	G	H	I	Total
Kim (2014)	√		√		√			√	√	5
Wang (2012)	√	√	√		√	√	√	√	√	8
Fais (2012)	√	√			√	√		√	√	6
Gao (2007)	√	√			√			√		4
Kim (2007)	√		√		√	√	√	√	√	7
Ko (2006)	√		√		√	√				4
Sekido (2003)	√	√	√		√	√		√		6
Lee (2002)	√				√	√				3
Liu (1999)	√	√			√					3
Tian (1998)	√		√		√					3
Tang (1997)	√	√			√	√				4
Takeshige (1993)	√				√					2
Takeshige (1992)	√				√					2
Han (1985)	√				√					2

Studies fulfilling the criteria of: A: peer reviewed publication; B: detailed statement of acupuncture procedure; C: objective behavior test applied to classify responder or non-responder; D: blinded assessment of outcome E: statement of cut-off value for dividing responder or non-responder; F: notification of the ratio of responder or non-responder to the total; G: reproducibility of EA sensitiveness; H: compliance with animal welfare regulations; I: statement of potential conflict of interests

### Cut-off values for EA responses

Thirteen studies used the tail flick latency (TFL) to distinguish between EA responders and non-responders; one employed the paw pressure threshold [25]. However, the criteria used to separate responders and non-responders in terms of EA sensitivity were very different. In 10 studies, percentage changes in pain behavior (from baseline) were calculated: cut-off values in the responder ranged 10–150% increase in TFL or the pressure pain threshold, and those in the non-responders were 0–50%. Three studies defined responders as animals exhibiting statistically significant increases in TFL (*p* < 0.05) compared with baseline [20, 21, 28]. One study defined a non-responder as an animal in which the TFL change was less than the mean value plus three standard deviations [24]. All criteria presented were shown in Table 3, and the proportion of responders according to the various criteria are shown in Additional file 1: Fig. S1, in the case of provided information available.

**Table 3:** EA response criteria and associated data

Author (Year)	Behavior test for cut off	EA frequency	EA response criteria: Changes from the baseline^a^ (*n*, % = proportion of responder or non-responder)
Kim et al. (2014)	TFL	LF	
Wang et al. (2012)	TFL	LF	
		HF	
Kim et al. (2007)	TFL	LF	
Ko et al. (2006)	TFL	HF	
Lee et al. (2002)	TFL	LF	
Liu et al. (1999)	TFL	HF	
Tian et al. (1998)	TFL	HF	
Tang et al. (1997)	TFL	HF	
Han et al. (1985)	TFL	IF	
Sekido et al. (2003)	PPT	LF	
Gao et al. (2007)	TFL	LF	
Takeshige et al. (1993)	TFL	LF	
Takeshige et al. (1992)	TFL	LF	
Fais et al. (2012)	TFL	LF	

^a^EA response criteria were differently applied. Researchers assessed with percentage change, *p* values or standard deviation: a) percentage change of during-EA or post-EA from the baseline, a’) converted into percentage change after direct contact to the author b) responder = significantly increase (*p* < 0.05 or *p* < 0.01) versus baseline, Non-responder = the others, c) Non-responder = post-EA TFL was less than baseline TFL + 3SD (*p* = 0.0014). HF, high frequency; *HR* high-responder, *IF* intermediate frequency, *LF* low frequency, *LR* low-responder, *n.a.* not applicable because of insufficient record, *NR* non-responder, *PPT* paw pressure threshold in normal rats, *R* responder, *SD* standard deviation, *TFL* Tail Flick latency

### Reproducibility of responsiveness to the anti-nociceptive or analgesic effects of EA

Wang et al. found that EA variance was maintained when TFL was evaluated on two successive days in a physiological state [32]. Kim et al. confirmed that responders exhibiting normal EA-mediated anti-nociception were consistently more sensitive to EA-induced analgesia in a model of neuropathic pain than were non-responders [18]. However, Sekido et al. reported different findings: 50% of normal rats were EA-responders, but all animals (thus both responders and non-responders) were susceptible to EA-induced analgesia after induction of carrageenan-mediated inflammatory pain [25].

### Intrinsic factors associated with a poor response to acupuncture

Six studies suggested that the level of CCK or the receptor thereof were related with no or a low response. Han et al. showed that injection of an antibody against the CCK-octapeptide (CCK-8) changed an EA non-response into an apparent response [19], and Tang et al. confirmed that inhibition of brain CCK synthesis rendered non-responders responders [30]. Liu et al. showed that the spinal perfusates of non-responders exhibited a higher level of CCK immunoreactivity than did that of responders [26]. Lee et al. found that CCK A receptor-encoding mRNA was highly expressed in the hypothalamus of non-responders and suggested that not only the CCK A receptor; CCK B receptor level per se but also that of the CCK receptor played important roles in the insensitivity to pain modulation afforded by EA [17]. Further, Ko et al. showed that the mRNAs encoding both the CCK A and CCK B receptor mRNAs were more highly expressed; these results were in contrast to those reported by Lee et al.. The use of different EA frequencies may explain these discrepancies, as Lee et al. delivered EA at 2 Hz, and the EA of Ko et al. was delivered at 100 Hz. Citing the data of Lee et al., Kim et al. found that CCK A receptor-knockout rats enjoyed significantly higher levels of anti-nociceptive effects after 2 Hz EA. Interestingly, the CCK receptor was more highly expressed in the hypothalamus of the non-responder group [17, 18, 29]. However, Wang et al. found no difference in the level of CCK receptor expression in the spinal dorsal horns of responders and non-responders [32] (Tables 4 and 5).

**Table 4:** Differentially expressed or changed factors between responder and non-responder

Author (Year)	Target regions	Responder > Non-responder	Non-responder > Responder
Low Frequency EA			
Kim (2014)	Hypothalamus	AMPK mRNA expression	
Wang (2012)^a^	Spinal dorsal horn (L5-L6)	- Neurotransmitter receptors-related genes: Aplnr, Gabrg2 (at 24 h) - Regulation of proinflammatory cytokines-related genes: Fcgr2b and Gsk3b	- Neurotransmitter receptors-related genes: Gabrg2 (at 1 h), and Htr1f - Proinflammatory cytokines-related gene expression: C5ar1, IL-6 and TNFα - Neurotransmitter receptors-related genes: Gabra2
Gao (2007)^b^	Hypothalamus	Voltage-gated K+ channels, solute carrier family 8, Synaptic vesicle glycoprotein 2b, glutamatergic A receptor, ghrelin precursor, melanocortin 4 receptor and neuroligin 1	
Lee (2002)	Hypothalamus		CCK-AR mRNA expression, but not CCK-BR
Takeshige (1993)	Dorsal PAG	Neural activity evoked by EA	
Takeshige (1992)	Medial arcuate nucleus of hypothalamus	Neural activity evoked by EA	
High Frequency EA			
Wang (2012)^a^	Spinal dorsal horn (L5-L6)	Aplnr, Gabrg2 (at 24 h)	None
Ko (2006)	Hypothalamus		Both CCK-AR and CCK-BR mRNA expression
Liu (1999)	Spinal perfusate		CCK-8-ir

^a^Through cDNA microarray, Wang (2012) compared the responder group, the non-responder group and the restraint group (not applied EA) at 1 h and 24 h time point after EA stimulation. We selectively reported genes which were significantly different between the responder group and the non-responder group and a more different group from the restraint group was described as the subject. Almost all genes, cited in this table, showed statistical difference at 1 h time point and only Gabrg2 was statistically different between the responder and the non-responder group both at 1 h and at 24 h time point. ^b^We selectively reported statistically different genes through both microarray and RT-PCR. Gao (2007) conducted dissecting hypothalamus immediately after EA stimulation. AMPK, 5′-AMP-activated protein kinase (regulation of energy homeostasis); CCK-8-ir, cholecystokinin octapeptide like immunoreactivity; CCK-AR, cholecystokinin A receptor; CCK-BR, cholecystokinin B receptor; PAG, periaqueductal central gray

**Table 5:** Biological factors that convert the EA response from responder to non-responder or vice versa

Author (Year)	EA	Factors	
		Responder → Non-responder	Non-responder → Responder
Kim (2014)	LF	Inhibiting AMPK activity in the hypothalamus	
Fais (2012)	LF		Inhibitor of norepinephrine and serotonin uptake at spinal terminals of descending pain inhibitory pathways (amitriptyline, i.p. or i.t)
Kim (2007)	LF		CCK-AR KO
Sekido (2003)	LF	Naloxone (opioid receptor antagonist, i.p.)	
Takeshige (1993)	LF	-Hypophysectomy -Antiserum of β-endorphin (i.c.v.)	Inhibitor of the degrading enzymes of met-enkephalin (DPA, i.p.)
Takeshige (1992)	LF	Hypophysectomy	- Morphine (i.p.) - Morphine (into post. Arcuate nucleus) - β-endorphin (into post. Arcuate nucleus)
Han (1985)	IF		CCK-8 AS (i.c.v. or i.t.)
Tian (1998)	HF	OFQ-Ab (i.t.)	OFQ-Ab (i.c.v.)
Tang (1997)	HF		Antisense CCK (i.c.v.)

*AMPK* 5′-AMP-activated protein kinase (regulation of energy homeostasis), *AS* antiserum, *CCK* cholecystokinin, *CCK-8* cholecystokinin octapeptide, *CCK-AR* cholecystokinin A receptor, *DPA* D-phenylalanine, *i.c.v.* intracerebroventricular injection, *i.p.* intraperitoneal injection, *i.t.* intrathecal injection, *KO* knockout, *Met-Enk* methionine enkephalin, *OFQ-Ab* orphanin FQ anibody, *post.* Posterior

### Intrinsic factors affording a good response to acupuncture

Several intrinsic factors were significant in EA responders. Takeshige et al. showed that the evoked potential in the posterior arcuate nucleus of hypothalamus and the dorsal periaqueductal gray matter of responders differed from that of non-responders [20, 21]. They also reported that administration of morphine (i.p. or microinjection into posterior arcuate nucleus of hypothalamus) increased the anti-nociceptive effects of EA to a level similar to high responders [20], whereas acupuncture- induced anti-nociception in responders remained unchanged. Both modulation of met-enkephalin [21] and β-endorphin [20, 21] could also convert the response of EA.

Hypophysectomy abolished the EA induced anti-nociception and evoked potentials of the posterior hypothalamic arcuate nucleus in responders (Tables 4 and 5) [20, 21]. Sekido et al. confirmed that an opioid receptor antagonist, naloxone (i.p.), attenuated EA analgesia in responders (Table 4) [25]. Fais et al. showed that EA combined with inhibition of norepinephrine and serotonin uptake (i.p. and i.t.) converted non-responders into responders (Table 4) [24]. Kim et al. found that hypothalamic 5′-AMP-activated protein kinase (AMPK) gene expression was upregulated after low-frequency EA in responder rats, and hypothalamic microinjection of a dominant-negative AMPK adenovirus, which inhibits AMPK activity, reduced EA analgesia; the wild-type control virus did not do so (Tables 4 and 5) [31].

Additionally, Sekido et al. demonstrated that peripheral inflammation potentiated the EA-induced sensitivity to analgesia; EA responders were about 50% as sensitive to EA analgesia in normal state, but all rats suffering from inflammation were more sensitive to EA. Naloxone, an opioid receptor antagonist, inhibited EA antinociception in normal rats but decreased EA analgesia in rats with inflamed paws in this study (Table 5) [25].

### Intrinsic factors influencing both high- and low-level responsiveness to acupuncture

Orphanin FQ (OFQ) influenced the response levels depending on the sites of action; intrathecal injection of anti-OFQ antibody converted responders into non-responders, and intracerebral injection of the antibody converted non-responders into responders (Table 5) [27].

### Candidate genes affecting EA response from transcriptomic analyses

Transcriptomic analyses revealed that gene expression levels in both the dorsal spinal cord and the hypothalamus varied among animals [28, 32]. Wang et al. profiled gene expression in the spinal dorsal horn after application of 2- and 100-Hz EA and found that the levels of mRNAs encoding neurotransmitter receptors (including the Aplnr, GABAA, glycine, and 5-HT1 receptors) were upregulated in responder (but not non-responder) rats [32]. They also showed that genes involved in inflammatory modulation were differentially expressed. In non-responder rats, mRNAs encoding proteins associated with the release of proinflammatory cytokines (e.g., IL-6 and TNF-α) were more highly expressed in non-responders than in responders after 2-Hz EA. However, genes inhibiting the release of proinflammatory cytokines, including Fcgr2b, GSK3b, and Tsc22d3, were upregulated to a greater extent in responders than in non-responders (Table 4) [32].

Gao et al. observed the changes of hypothalamic gene expressions using microarray analysis. They showed that that several genes including a glutamatergic receptor (Grm6), a precursor of ghrelin (Ghrl), the melanocortin 4 receptor (Mc4r), and neuroligin 1 (Nlgn1) were significantly upregulated in the hypothalamus of responders (Table 4) [28].

## Discussion

**Fig. 2:**
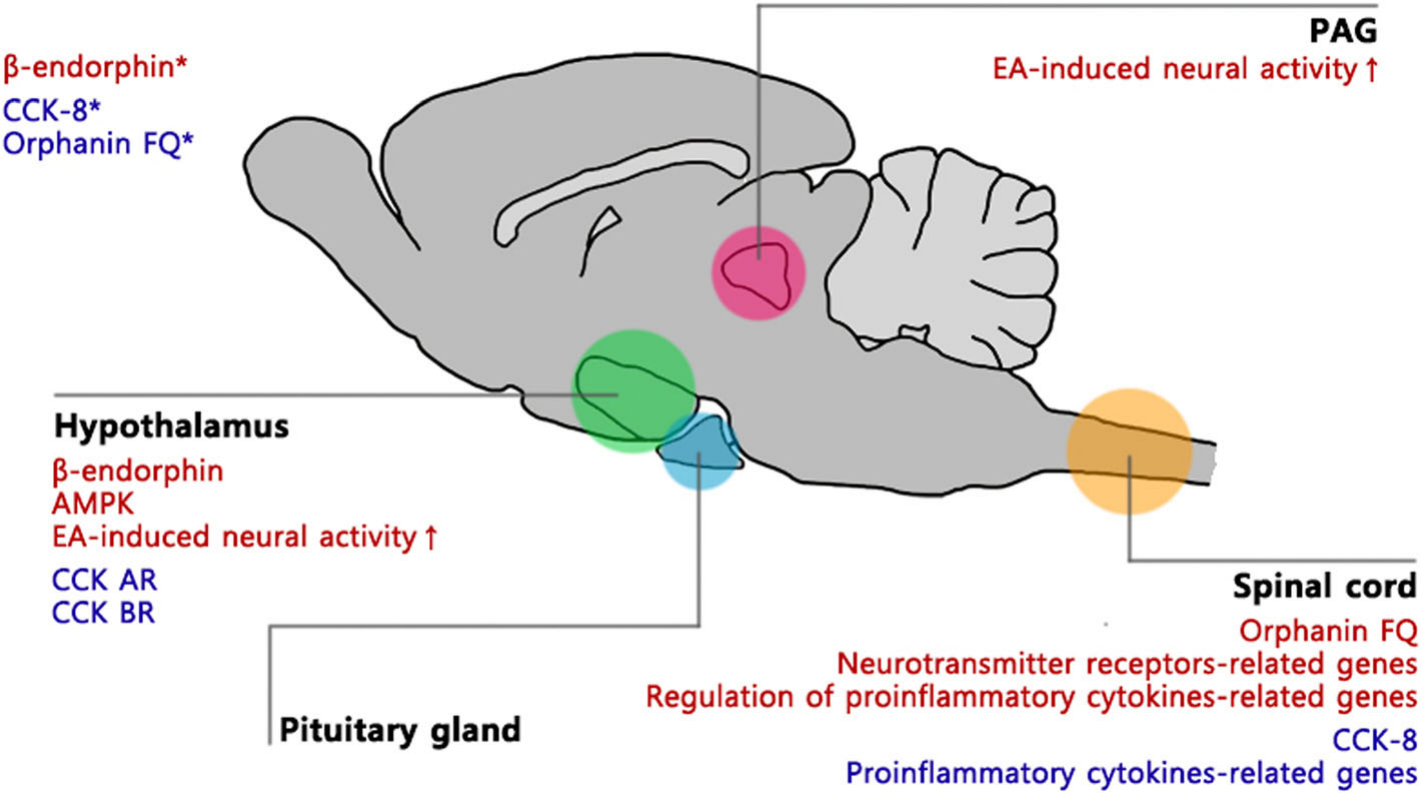
A scheme of EA responsiveness-related factors and regions. The indicated specific regions, hypothalamus, PAG, pituitary gland and spinal cord, are well-known to be associated with pain regulation, and have been reconfirmed through researches on EA responsiveness. An asterisk implies intracerebroventricular injection. Responder-related factors and non-responder-related factors have been grouped in red and blue letters respectively. CCK AR, cholecystokinin A receptor; CCK BR, cholecystokinin B receptor; CCK-8, cholecystokinin octapeptide; Hypo, hypothalamus; PAG, periaqueductal grey; Pit. g., pituitary gland

In this review, we have shown that the levels of signaling molecules associated with acupuncture analgesia (those of the descending inhibitory system, endogenous opioids, and CCK-8 and receptors thereof) may be differentially expressed in responders and non-responders. Also, modulation of such factors may change the response to EA. Responders and non-responders differ in terms of AMPK expression in the hypothalamus and in the levels of neurotransmitter receptors and pro-inflammatory cytokines genes in the spinal cord [31, 32] and such differences may also affect response related to EA (Fig. 2).

Acupuncture analgesia is an integrative process involving both afferent impulses from the acupoints and the painful region [12]. In an early responder study, Takeshige et al. suggested that afferent neural transmission could explain different responses to EA [20, 21]. Upon application of EA, the changes in the neuronal activity in the arcuate nucleus and dorsal PAG differed between responders and non-responders and correlated with the extent of EA-induced analgesia. EA increased neuronal activity in the PAG of responders, irrespective of whether EA was applied ipsilaterally or contralaterally.

As mentioned above, the endogenous descending inhibitory system of the central nervous system (CNS) and various signaling molecules, including opioid peptides, glutamate, and 5-hydroxytryptamine, contribute to acupuncture-induced analgesia [33]. The release of opioid peptides during acupuncture is frequency-dependent. Low- and high-frequency EA release enkephalin/β-endorphin and dynorphin, respectively, in the CNS [34]. Responder analysis has also shown that differences in the expression levels of these factors contribute to individual differences in sensitivity to EA analgesia. Sekido et al. found that intraperitoneal injection of naloxone converted responders to low-frequency EA into non-responders [25]. Injection of anti-β-endorphin antibody into the cerebral ventricles abolished the analgesic effects of low-frequency EA in responders (21). D-phenylalanine, an inhibitor of enzymes degrading met-enkephalin, morphine, and β-endorphin changed the neuronal activity induced by low-frequency EA in non-responders to that characteristic of responders [20, 21]. The OFQ peptide is involved in many physiological processes, including pain. The effects of the peptide on the nervous system are complex; it is generally accepted that spinal OFQ is anti-nociceptive, while OFQ exerts an anti-opioid action and causes hyperalgesia when injected supraspinally [35]. Tian et al. explored whether OFQ modulated the responses of EA and found that intrathecal injection of an anti-OFQ antibody converted responders to non-responders, whereas intracerebral injection had the reverse effect [27].

Additionally, the norepinephrine and serotonin systems of the descending inhibitory pathway contribute to individual differences in EA analgesia. Fais et al. found that the poor analgesia of non-responders changed to the level of analgesia enjoyed by responders after administration (i.p. and i.t.) of inhibitors of norepinephrine and serotonin uptake at the spinal terminals of the descending inhibitory pathways [24].

Kim et al. suggested that hypothalamic AMPK might be an intrinsic mediator of the EA response [31]. The AMPK enzyme plays roles in cellular energy homeostasis and metabolic stress and was recently shown to modulate both acute and chronic neuropathic pain [36, 37]. Hypothalamic AMPK gene expression was upregulated after low-frequency EA in responder rats, and hypothalamic inhibition of AMPK activity using dominant-negative AMPK adenovirus reduced EA analgesia.

The best-studied intrinsic factors are CCK-8 and receptors thereof. CCK is a CNS neurotransmitter playing roles in pain, satiety, feeding, learning, cognition, and emotion. CCK-8, a major form of CCK, is found predominantly in the CNS [38–40], and has been found to play a role in the development of tolerance to EA; CCK-8 may counter pain alleviation by exerting an anti-opioid action [41]. Liu et al. showed that the extent of CCK-8 immunoreactivity increased in the spinal perfusate of non-responders after high-frequency EA [26]. Intracerebroventricular or intrathecal injection of anti-CCK-8 antibody or antisense CCK-8 converted non-responders into responders [19]. However, the levels of CCK in the spinal dorsal horn were increased in both responders and non-responders after EA [32]. It has been suggested that the CCK receptor isoforms, CCK AR and BR are differentially affected [29]. However, it remains unclear which receptor may be more important in terms of the response to EA analgesia. Upon application of low-frequency EA, the level of CCK AR but not BR mRNA increased [17]. CCK AR-knockout rats exhibited an elevated anti-nociceptive response after low-frequency EA [18]. In contrast, the hypothalamic levels of mRNAs encoding both CCK AR and BR were upregulated after high-frequency EA. A CCK-B antagonist potentiated EA anti-nociception after high-frequency EA [42]. However, no significant changes in the levels of CCK AR or BR mRNAs were evident in the dorsal horn of the spinal cord [32]. Thus, it seems clear that the CCK system reduces the analgesic effects of EA, regardless of the frequency. However, further research is required to determine whether this is the principal cause of EA non-responsiveness.

Transcriptomic analyses have suggested that the actions of several novel intrinsic factors may explain the different responses to acupuncture. Wang et al. found that the differential regulation of genes encoding neurotransmitters and their receptors indicated that the neurotransmitter system may be more active in responders than in non-responders. Genes involved in the modulation of inflammation were also differentially expressed. In non-responder rats, the levels of mRNAs facilitating the release of proinflammatory cytokines (including IL-6 and TNF- α) were higher in non-responders. In responders, the levels of mRNAs inhibiting the release of proinflammatory cytokines (e.g., Fcgr2b, GSK3b, and Tsc22d3) were higher in responders. It is known that proinflammatory cytokines induce the release of various inflammatory materials and play important roles in the nociceptive and analgesic systems of the CNS. These results suggest that the intrinsic response to spinal cord inflammation may partly explain the different responses to EA [32].

There are several issues to discuss for developing the responder analysis more useful. First, the reproducibility of the EA response is an important consideration. If factors influencing this response are intrinsic, the response must be reproducible. Wang et al. repeated TFL tests on the same rats at 2-day intervals and found that the EA response was maintained [32]. However, most studies have not addressed reproducibility. Additionally, more than 70 % of the included studies measured anti-nociceptive effects in non-diseased normal animals, and it is doubtful that results can be applicable to diseased individuals. Kim et al. explored whether the anti-nociceptive response to EA in non-diseased normal animals was reproduced after induction of neuropathic pain; the responses of both responders and non-responders were in fact maintained [18]. However, Sekido et al. reported a different finding: 50% of non-diseased normal rats were classified as EA responders, but all tested rats, regardless of response status, experienced EA-induced analgesia of carrageenan-induced inflammatory pain [25]. In addition, it should be considered that the ratio of responder and non-responder might be varied across the types of disease. In Li et al.’s study, the number of responder was twice as many as that of non-responder for the modulatory effects of EA (acupoint PC5-PC6) on cardiovascular reflex response [43], while there are not enough data for the responder ratio in other physiological or pathological condition. Further studies with different pathological models should address this question.

Second, the classification of responders or non-responders needs to set a good validation. The cut-off criteria for responders were extremely variable among the 14 relevant studies, including a 10–150% increase in the TFL or the pressure pain threshold from baseline. The notifying accurate description of classifying responder or non-responder and the use of validated cut-offs is essential in future work. Next, the acupuncture conditions reviewed are very limited; all studies employed very common methods such as EA of acupoint ST36 (100% of studies). There are more than 360 acupoints with various stimulation methods [44]. Thus, caution is required when generalizing the results.

Further, the responder studies in clinical setting are limited. Only a few studies have sought to identify human responders. Chae et al. used a microarray to identify genes differing in expression level between high- and low-responders in 15 healthy volunteers. Genes related with signaling, stress response, transcriptional regulators, and/or regulators of immune function may be relevant, although the “responder” mechanism needs to be clarified further [45]. Genetic factors have been suggested to play roles in individual sensitivity to acupuncture [5, 13, 45]. However, the gap between pre-clinical and clinical researches remains very wide, and further translational clinical studies are essential.

Next, various parameters beyond individual variance can affect the responsiveness of acupuncture. For instance, the selection of acupoints, the number of acupuncture treatments, stimulation modality (manual, EA or others) and intensity, learning from pre-exposed cues, and the condition of the patients can influence the acupuncture effects [34, 46–50]. EA tolerance, which means a gradual decline of EA effects due to the repeated use, is also important to explain the low-responsiveness of EA [51]. It might be interesting how these various factors synergistically contribute to acupuncture effects.

Finally, it is also important to consider how the various intrinsic factors identified can contribute to the elucidating the acupuncture mechanism. Though there are still lots of limitation, the existed evidences suggest this possibility: the factors identified in the low responder (i.e. CCK-8 and spinal OFQ) have shown to be involved in the attenuation of EA analgesia and the induction of EA tolerance [19, 26, 27]; the injection of morphine and β-endorphin could convert non-responder into high-responder, and these results are helpful to understand the involvement of endogenous opioid system in the acupuncture analgesia [20, 21]. Further well designed responder analysis is needed to aid the acupuncture mechanism.

In spite of the several limitations mentioned above, our review of pre-clinical studies is meaningful to show the benefit of responder and non-responder analysis for elucidating the mechanism of acupuncture in pain. Further responder analysis considering the validated cut-off criteria, animal models depicting human disease, and real-world acupuncture methods is recommendable. This kind of approach might help to develop the strategy to enhance the acupuncture effects in pain medicine.

## Conclusion

In conclusion, we confirmed that dividing animals into responders and non-responders identified novel candidate mediators of the effects of acupuncture. A number of studies have found that the EA responsiveness could be modifiable by modulating these mediators. Although most included studies regarding EA responsiveness was investigated in the physiological state, and there are several methodological issues to be improved, this study may allow us the development of new strategies potentiating the therapeutic effects of acupuncture.

## Additional file

Additional file 1: Fig. S1. Varied ratio of responders according to arbitrary cut-off value. Low frequency EA was arranged on the left and high frequency EA on the right. Since an arbitrary cut-off value can change the allocation of responder rats, we divided absolutely high responsiveness as a genuine responder and relatively high responsiveness as a modifiable responder. This allowed those with relatively high responsiveness not to be misallocated as a responder with a higher cut-off value. (PNG 847 kb)

## Abbreviations

AMPK: 5′-AMP-activated protein kinase
CCK AR: cholecystokinin A receptor
CCK BR: cholecystokinin B receptor
CCK: cholecyctokinin
CCK-8: cholecystokinin octapeptide
CNS: central nervous system
EA: electroacupuncture
OFQ: Orphanin FQ
PAG: periaqueductal grey
TFL: tail flick latency
