# Supplementary figures and images for: What intrinsic factors influence responsiveness to acupuncture in pain?: a review of pre-clinical studies that used responder analysis
Source: BMC Complement Altern Med. 2017 May 25;17:281. doi: 10.1186/s12906-017-1792-2 (PMC5445410; doi:10.1186/s12906-017-1792-2)

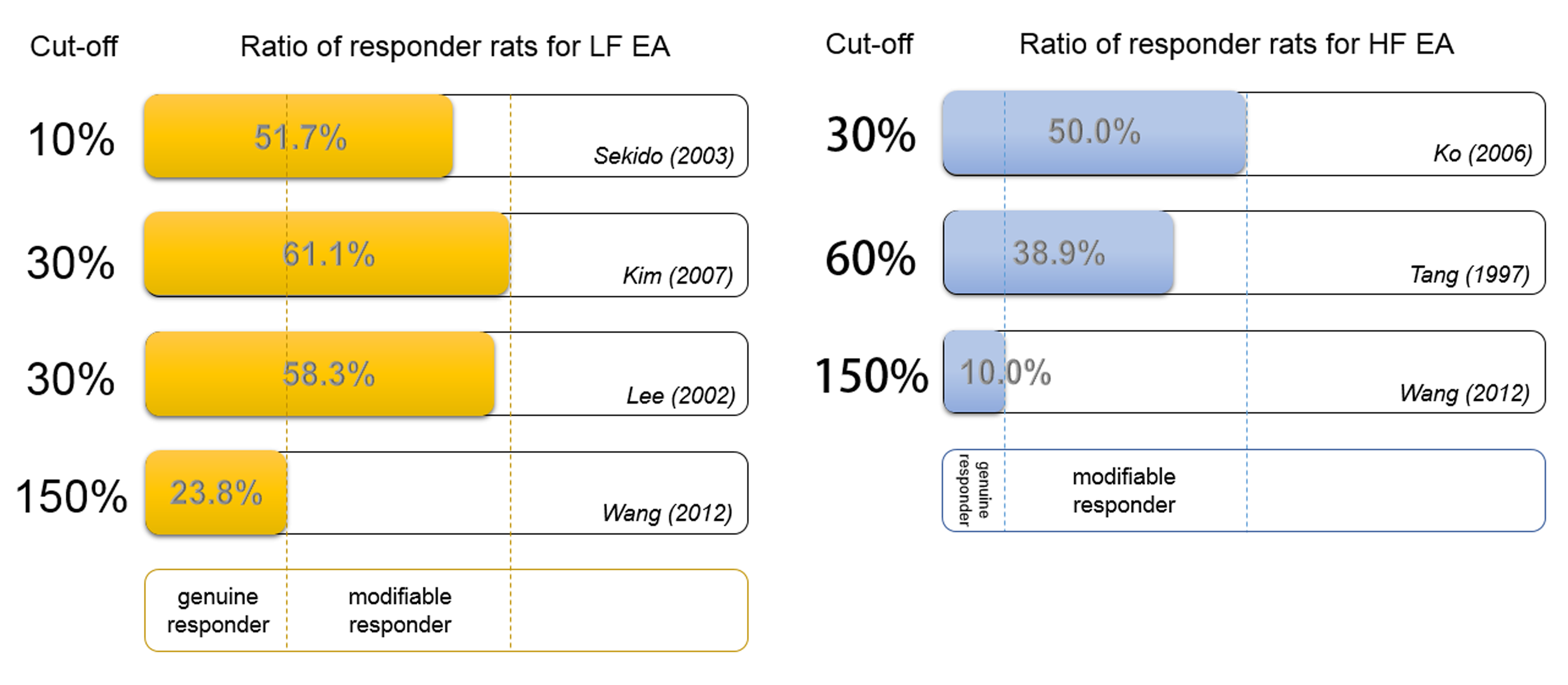

Supplement: Additional file 1: Fig. S1. — Varied ratio of responders according to arbitrary cut-off value. Low frequency EA was arranged on the left and high frequency EA on the right. Since an arbitrary cut-off value can change the allocation of responder rats, we divided absolutely high responsiveness as a genuine responder and relatively high responsiveness as a modifiable responder. This allowed those with relatively high responsiveness not to be misallocated as a responder with a higher cut-off value. (PNG 847 kb) [file 12906_2017_1792_MOESM1_ESM.png]
